# Screening of an FDA-approved compound library identifies apigenin for the treatment of myocardial injury

**DOI:** 10.7150/ijbs.85204

**Published:** 2023-10-16

**Authors:** Haixia Li, Dong Chen, Xiaoqin Zhang, Mingxian Chen, Yinghao Zhi, Weilu Cui, Shanshan Li, Fan Xu, Ying Tan, Hao Zhou, Xing Chang, Hengwen Chen

**Affiliations:** 1 Guang'anmen Hospital, China Academy of Chinese Medical Sciences, Beijing, 100053, China.; 2 Dongzhimen Hospital of Beijing University of Traditional Chinese Medicine, Beijing, 100000, China.; 3 Beijing University of Chinese Medicine, Beijing, 100029, China.; 4 Zhejiang Tongde Hospital, Hangzhou, 310012, China.; 5 Wenzhou TCM Hospital of Zhejiang Chinese Medical University, Wenzhou, 325000, China.; 6 School of Medicine, Southern University of Science and Technology (SUSTech), Shenzhen, Guangdong, China.

**Keywords:** apigenin, doxorubicin, mitochondrial unfolded protein response, Sirt1, Atf5, cardiomyopathy

## Abstract

Apigenin is the active ingredient in Ludangshen. Although previous studies reported the cardioprotective actions of apigenin against doxorubicin (Dox)-induced cardiomyopathy, the underlying mechanisms remain incompletely understood. Since apigenin beneficially regulates various aspects of mitochondrial function and dynamics, we asked whether apigenin improves heart function in mice with Dox-induced cardiomyopathy by regulating the mitochondrial unfolded protein response (UPR^mt^). Co-administration of apigenin significantly restored heart function, reduced myocardial swelling, inhibited cardiac inflammation, increased cardiac transcription of UPR^mt^-related genes, and promoted cardiomyocyte survival in Dox-treated mice. In turn, blockade of UPR^mt^ abolished the mito- and cytoprotective effects of apigenin, evidenced by decreased ATP production, suppressed mitochondrial antioxidant capacity, and increased apoptosis, in Dox-treated, cultured HL-1 cardiomyocytes. Furthermore, apigenin treatment prevented Dox-induced downregulation of Sirt1 and Atf5 expression, and the beneficial effects of apigenin were completely nullified in *Sirt1* knockout (KO) mice or after siRNA-mediated *Sirt1* knockdown *in vitro*. We thus provide novel evidence for a promotive effect of apigenin on UPR^mt^ via regulation of the Sirt1/Atf5 pathway. Our findings uncover that apigenin seems to be an effective therapeutic agent to alleviate Dox-mediated cardiotoxicity.

## Introduction

Doxorubicin (Dox), a member of the anthracycline class of antineoplastic drugs, is widely used as a chemotherapeutic agent in patients with breast cancer, lymphomas, sarcomas, and other tumors. However, its clinical application is limited by common cardiotoxic effects, including decreased cardiac output, microvascular occlusion, myocardial fibrosis, and even cardiomyocyte death 1, 2. Several mechanisms, including reactive oxygen species (ROS) overloading, DNA damage, an excessive inflammatory response, metabolic reprogramming, and abnormal autophagy, have been proposed to explain Dox-related cardiotoxicity 3-5. However, despite much efforts, a complete picture of the key signaling pathways involved has not yet emerged. Accordingly, there is still a lack of drugs that effectively prevent or ameliorate myocardial damage and preserve heart function in patients treated with Dox.

The mitochondrial unfolded protein response (UPR^mt^) is a conserved mitochondrial protective mechanism that controls the nuclear transcription of antioxidant, antiapoptotic, and antiinflammatory genes 6, 7. Previous studies have identified the beneficial actions of the UPR^mt^ in septic cardiomyopathy 8 and cardiac ischemia reperfusion injury (IRI) 9. It was further demonstrated that UPR^mt^ activation reduces mitochondrial ROS production and inhibits mitochondria-dependent cardiomyocyte death 10-13. In addition, augmented UPR^mt^ activity is associated with increased mitophagy, which also serves to sustain mitochondrial performance 14. However, whether detrimental regulation of the UPR^mt^ contributes to Dox-induced cardiotoxicity remains unclear.

Apigenin is the active ingredient in Ludangshen, and present in a wide variety of fruits and vegetables 15-17. Previous studies have reported that apigenin protects endothelial function against oxidative stress during vascular inflammation 18. In turn, an anti-fibrosis effect of apigenin in isoproterenol-induced cardiomyopathy was attributed to negative regulation of the Smad pathway 19. In a rat model of LPS-induced endotoxemia, administration of apigenin prevented cardiomyocyte apoptosis by suppressing the SphK1/S1P pathway 20. In addition to the beneficial effects of apigenin on cardiovascular diseases, reported in animal models, several investigations elucidated also the protective role of apigenin against mitochondrial abnormalities. Thus, mitophagy 21, mitochondrial oxidative stress 22, mitochondrial biogenesis 23, and mitochondrial metabolism 24 have been proposed as downstream targets of apigenin. Based on the above information, we considered worthy to explore whether apigenin alleviates Dox-related cardiomyopathy by regulating the UPR^mt^.

The NAD-dependent deacetylase Sirtuin-1 (Sirt1) sustains mitochondrial function through affecting a range of mitochondria-related processes such as ROS production 25, mitophagy 26, and mitochondria-dependent cell death 27. Importantly, Sirt1 is reportedly an intracellular effector of several Traditional Chinese Medicine (TCM) bioactive agents, such as resveratrol 28, salidroside 29, and atractylenolide III 30. Since activating transcription factor 5 (Atf5) has been identified as an upstream inducer of the UPR^mt^ during cardiac IRI 9, we hypothesized that Sirt1 and/or Atf5 might be downstream targets of apigenin. Therefore, we performed animal experiments and cellular studies to examine whether apigenin confers protection against Dox-related cardiomyopathy through regulating the Sirt1/Atf5 pathway to influence the UPR^mt^.

## Results

### Apigenin attenuates Dox-mediated myocardial injury

**Figure 1:**
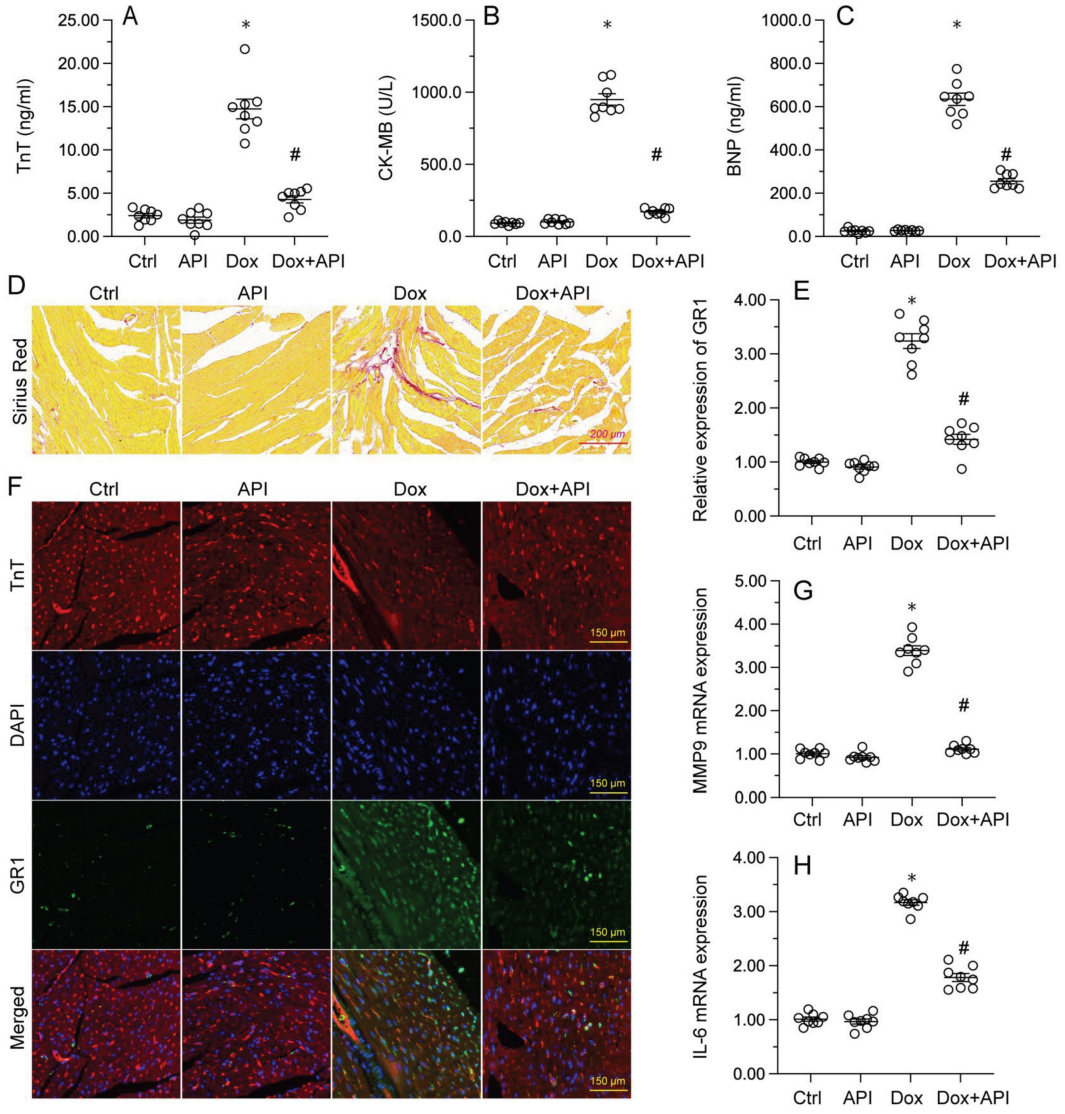
** Apigenin attenuates Dox-mediated myocardial injury.** Mice were treated with Dox (15 mg/kg) with or without oral apigenin co-administration (25 mg/kg) for 4 weeks. **(A-C)** Serum was isolated from mice and the levels of TnT, BNP, and CK-MB were measured through ELISA. **(D)** Sirius Red staining was used to observe histological alterations in heart tissue. **(E**,** F)** GR-1 immunofluorescence was used to evaluate neutrophil infiltration in the myocardium.** (G-H)** The transcription of IL-6 and MMP9 in heart tissues was assessed by RT-PCR. *p<0.05.

To determine the beneficial actions of apigenin on Dox-induced cardiomyopathy, apigenin was administered to Dox-treated mice 3 days before histological and molecular analyses. Cardiac injury was first assessed by measurements of serum TnT, BNP, and CK-MB levels using ELISA. Results showed that compared to control (PBS-treated) mice, the concentrations of these three markers were significantly increased after Dox treatment (Figure 1A-1C). In contrast, serum TnT, BNP, and CK-MB levels were markedly attenuated in mice treated with apigenin (Figure 1A-1C). Sirius Red staining was used to assess whether apigenin treatment alleviated also Dox-induced histological alterations in heart tissue. Consistent with the above results, myofibrillar fibrosis and swelling were noted in Dox-injected mice while these structural alterations were relieved by pretreatment with apigenin (Figure 1D). Meanwhile, GR-1 immunofluorescence indicated substantial accumulation of neutrophils in heart tissues from Dox-treated mice compared to PBS-treated mice. In contrast, decreased neutrophil accumulation, suggestive of a reduced inflammatory response, was observed after apigenin administration (Figure 1E and 1F). In addition, RT-PCR analysis showed that Dox treatment increased cardiac transcription of proinflammatory genes (IL-6 and MMP9) when compared with the control mice while the above alterations were significantly suppressed in the presence of apigenin treatment (Figure 1G-1H). These data indicated that apigenin effectively relieved Dox-mediated myocardial injury in mice.

### Apigenin attenuates cardiac dysfunction and normalizes cardiomyocyte function following Dox exposure

**Figure 2:**
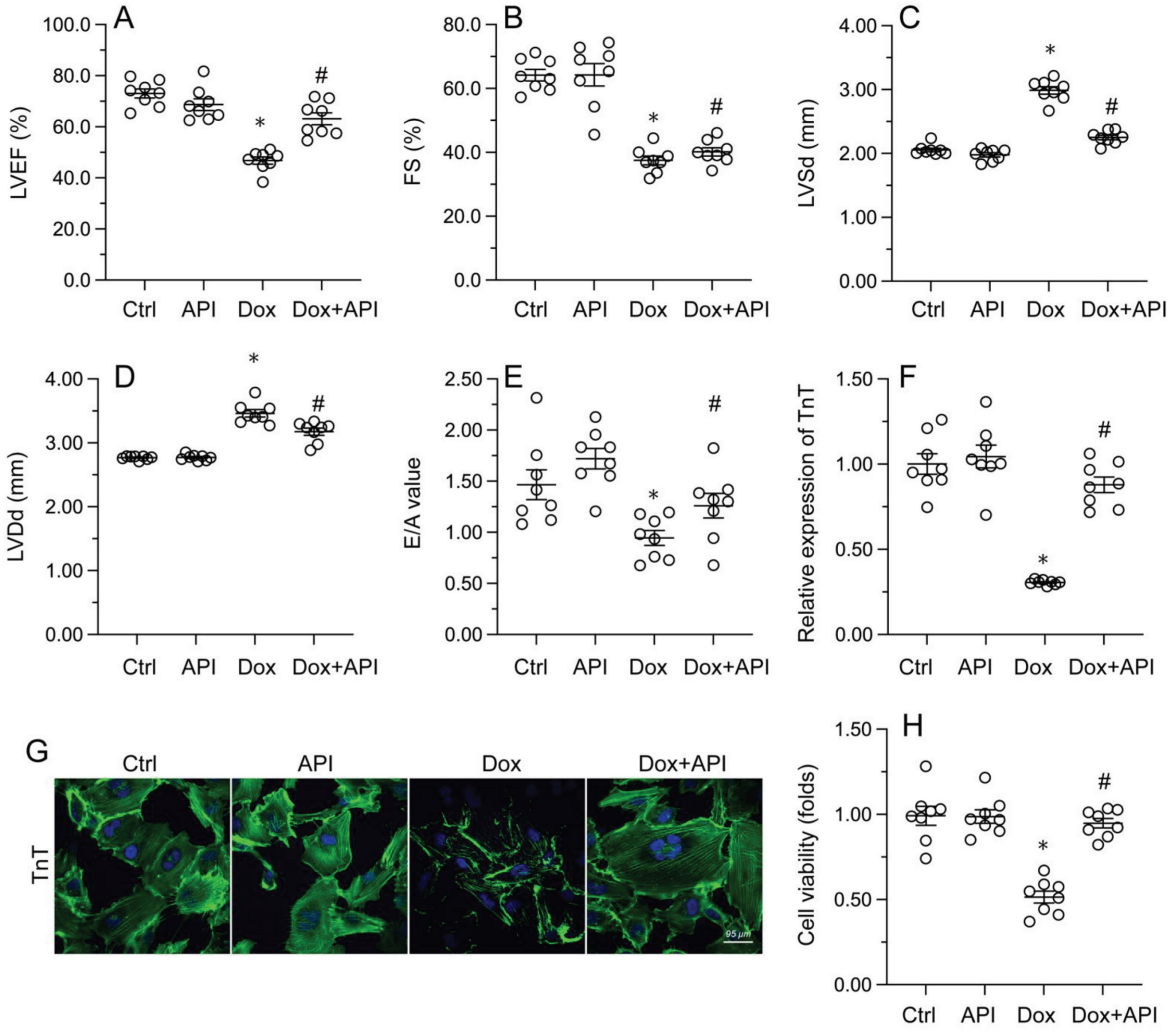
** Apigenin normalizes cardiomyocyte function in the presence of Dox. (A-E)** Echocardiographic analysis of heart function in mice. **(F, G)** TnT immunofluorescence in HL-1 cells treated with Dox (1 μM, 24 h) in the presence or absence of apigenin (50 μM). **(H)** The MTT assay was used to analyze HL-1 cell viability. *p<0.05.

Echocardiography was then used to confirm that apigenin treatment protected heart function in mice with Dox-induced cardiomyopathy. After Dox treatment, LVEF and FS were significantly downregulated, whereas E/A, LVDd, and LVSd were markedly augmented, relative to baseline (Figure 2A-2E). However, in apigenin-treated mice, systolic function (LVEF, FS, and LVSd) as well as diastolic capacity (LVDd, E/A) were partially restored to near-physiological condition (Figure 2A-2E). These results demonstrated that apigenin protects heart function in mice with Dox-induced cardiomyopathy.

To assess the molecular mechanisms by which apigenin protects heart function upon Dox exposure, mouse cardiac HL-1 cells *in vitro* were challenged with Dox alone or in combination with apigenin. Then, TnT immunofluorescence staining was applied to evaluate the contractile cytoskeleton. Compared with PBS-treated cells, reduced expression of TnT was observed after Dox exposure (Figure 2F and 2G). Suggesting a protective mechanism against Dox-related myocardial dysfunction, apigenin administration partially restored TnT expression in Dox-challenged HL1- cells (Figure 2F and 2G). Using the MTT assay, we also noted that cardiomyocyte viability was rapidly reduced upon Dox exposure, and this effect was markedly attenuated after co-administration of apigenin (Figure 2H). These results showed that apigenin sustains cardiomyocyte function by preventing TnT degradation, and supports also cardiomyocyte viability in the presence of Dox.

### Apigenin induces UPR^mt^ activation in Dox-exposed cardiomyocytes

**Figure 3:**
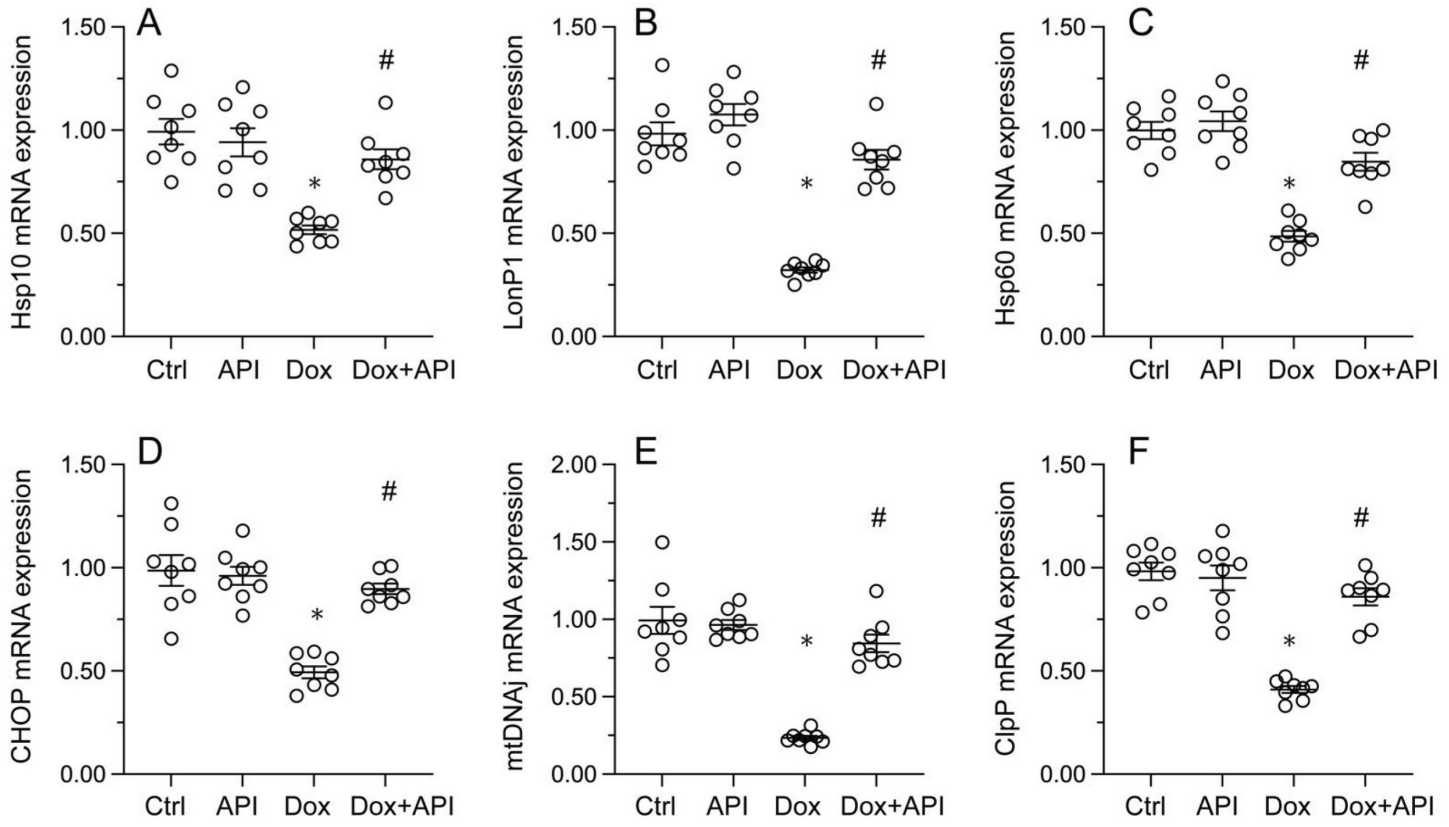
** Apigenin enhances the survival of Dox-exposed cardiomyocytes by inducing the UPR^mt^. (A-F)** RT-PCR was used to analyze the impact of apigenin treatment on the transcription of UPR^mt^-related genes in heart tissue from Dox-treated mice. *p<0.05.

To evaluate whether apigenin actions involve changes in UPR^mt^ activity in cardiac tissue exposed to Dox, RT-PCR assays were conducted to assess the transcription of UPR^mt^-related genes, namely *ClpP*, *mtDNAj*, *CHOP*, *Hsp10*, *Hsp60*, and *LonP1*. Data illuminated that the transcription of the corresponding mRNAs was rapidly depressed in heart tissues from Dox-treated mice, compared to PBS-treated animals (Figure 3A-3F). In turn, suggesting UPR^mt^ restoration, apigenin treatment counteracted Dox-mediated downregulation of the referred genes (Figure 3A-F).

### Apigenin-mediated UPR^mt^ activation sustains mitochondrial function and integrity in Dox-treated cardiomyocytes

**Figure 4:**
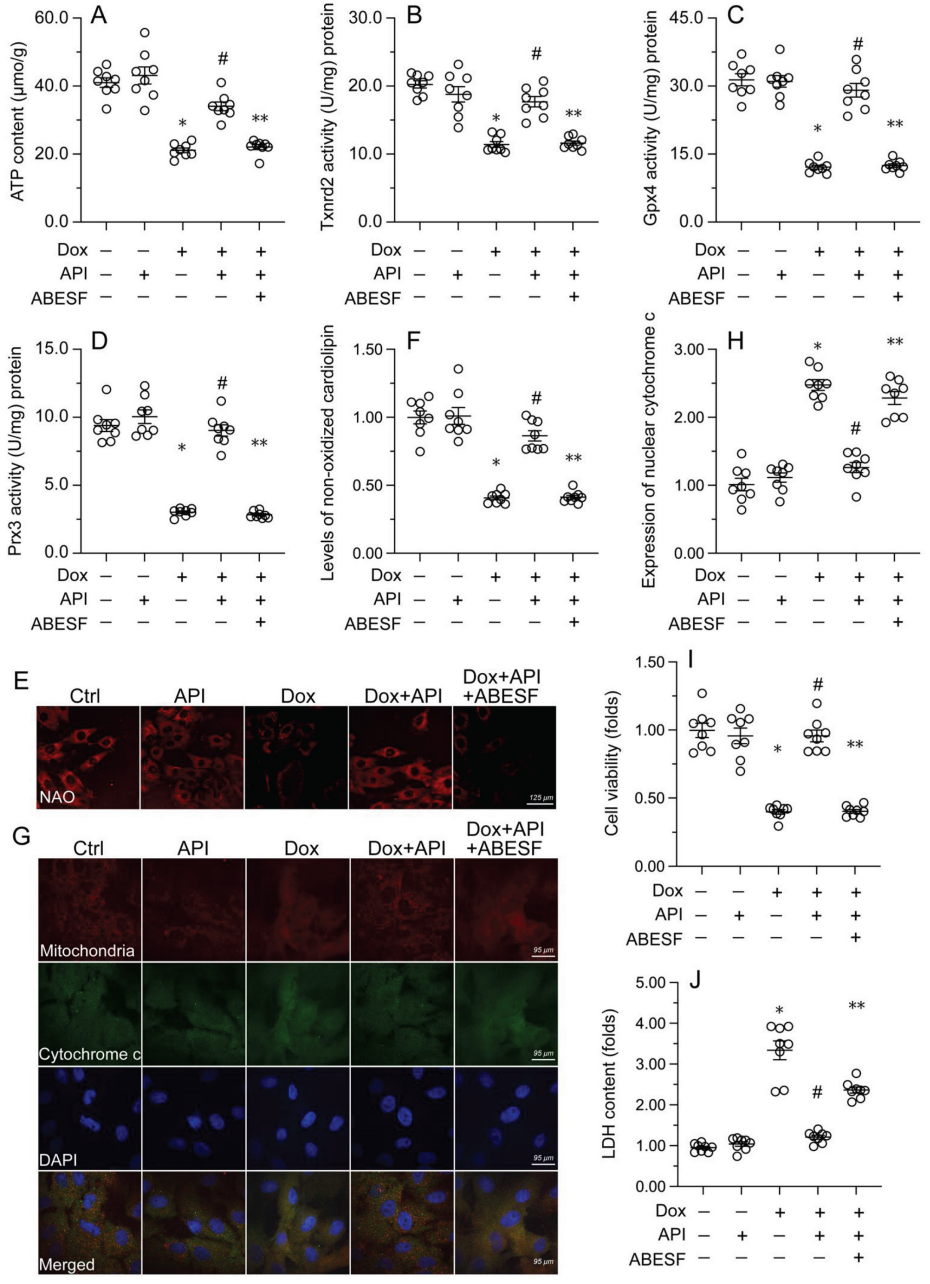
** Apigenin-mediated UPR^mt^ activation sustains mitochondrial function and integrity in Dox-challenged cardiomyocytes. (A)** ELISA was used to measure the concentration of ATP in cultured HL-1 cells. **(B-D)** ELISA-based analysis of the activity of mitochondria-localized antioxidant enzymes. **(E, F)** Immunofluorescence analysis of peroxidized cardiolipin levels. **(G, H)** Immunofluorescence detection of cytochrome c localization in cultured HL-1 cells. **(I)** The MTT assay was used to measure HL-1 cell viability. **(J)** The LDH release assay was used to evaluate cytotoxicity in HL-1 cells. ABESF (10 μM) was applied to cells 4 h before incubation with apigenin. *p<0.05.

To test the hypothesis that enhanced UPR^mt^ sustains mitochondrial function and integrity in Dox-exposed cardiomyocytes, mitochondrial function was first assessed by measuring ATP concentrations in HL-1 cells. ELISA results showed that Dox treatment repressed ATP production, whereas this change was reversed by apigenin (Figure 4A). However, reduced ATP synthesis was observed after co-administration of ABESF to prevent UPR^mt^ activation (Figure 4A). Similarly, apigenin treatment prevented the repression of mitochondria-localized antioxidant enzymes (i.e. Gpx4, Prx3, and Txnrd2) induced by Dox while these changes were was negated once treatment with ABESF (Figure 4B-4D). Due to decreased mitochondrial antioxidant capacity, the levels of peroxidized cardiolipin were significantly elevated in response to Dox treatment (Figure 4E and 4F). Notably, the content of non-oxidated cardiolipin was significantly restored in apigenin-treated cells, and this effect was also abrogated by ABESF (Figure 4E and 4F). Lastly, we observed that Dox treatment accelerated the translocation of cytochrome c from mitochondria into the cytoplasm or nucleus (Figure 4G and 4H). Although apigenin markedly maintained cytochrome c retention in the cytoplasm, this affect was obviously abolished in ABESF-treated cells (Figure 4G and 4H). These results confirmed that apigenin protects mitochondrial function and integrity in Dox-treated cardiomyocytes through stimulation of the UPR^mt^.

To further examine whether the UPR^mt^ is a protective mechanism in Dox-treated cardiomyocytes, ABESF, an inhibitor of the UPR^mt^, was co-applied to HL-1 cell cultures. MTT assay results showed that upon Dox exposure, ABESF pre-treatment reduced cardiomyocyte viability in the presence of apigenin (Figure 4I). In addition, LDH release assays showed that apigenin was able to prevent Dox-mediated LDH leakage, whereas this protective effect was nullified by ABESF (Figure 4J). These results indicated that upon Dox exposure, apigenin maintains cardiomyocyte viability and function through reversing the inactivation of the UPR^mt^.

### Apigenin activates the UPR^mt^ through the Sirt1/Atf5 pathway

**Figure 5:**
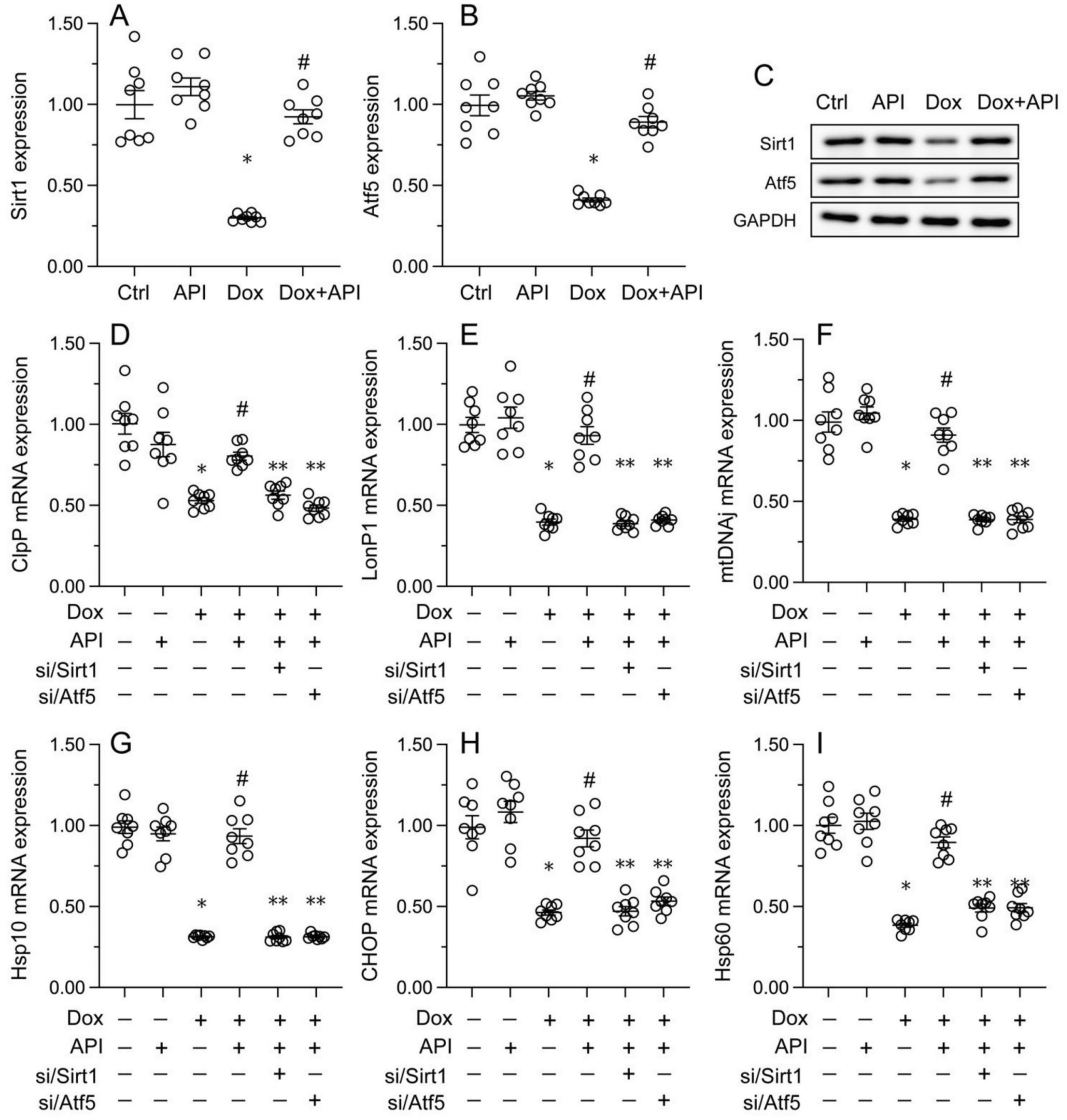
** Apigenin activates the UPR^mt^ through the Sirt1/Atf5 pathway.** siRNAs against Sirt1 (si/Sirt1) or Atf5 (si/Atf5) were transfected into HL-1 cells before treatment with Dox. **(A-C)** Western blot analysis of Sirt1 and Atf5 expression in HL-1 cells. **(D-I)** RT-PCR assays were used to analyze the transcription of UPR^mt^-related genes in HL-1 cells. *p<0.05.

To address the regulatory mechanism(s) by which apigenin activates the UPR^mt^ during Dox-induced cardiomyopathy, we focused on the Sirt1/Atf5 pathway. Protein analysis elucidated that the protein levels of both Sirt1 and Atf5 were obviously downregulated in Dox-treated compared to PBS-treated HL-1 cells. Interestingly, this change was reversed by apigenin administration (Figure 5A-5C).

To assess whether Sirt1/Atf5 pathway restoration underlies apigenin-mediated UPR^mt^ activation, siRNAs against Sirt1 (si/Sirt1) and Atf5 (si/Atf5) were alternatively transfected into HL-1 cells prior to Dox/apigenin treatment(s). Then, relevant UPR^mt^ markers were measured again. Upon Dox-treatment, apigenin failed to restore the transcription of *ClpP*, *mtDNAj*, *CHOP*, *Hsp10*, *Hsp60*, and *LonP1* in si/Sirt1- or si/Atf5-transfected HL-1 cells (Figure 5D-5I). These results therefore identify Sirt1/Atf5 pathway activation as the molecular mechanism by which apigenin elicits the UPR^mt^ in Dox-challenged cardiomyocytes.

### Sirt1 knockdown abolishes the cardioprotective effects of apigenin in Dox-treated mice

**Figure 6:**
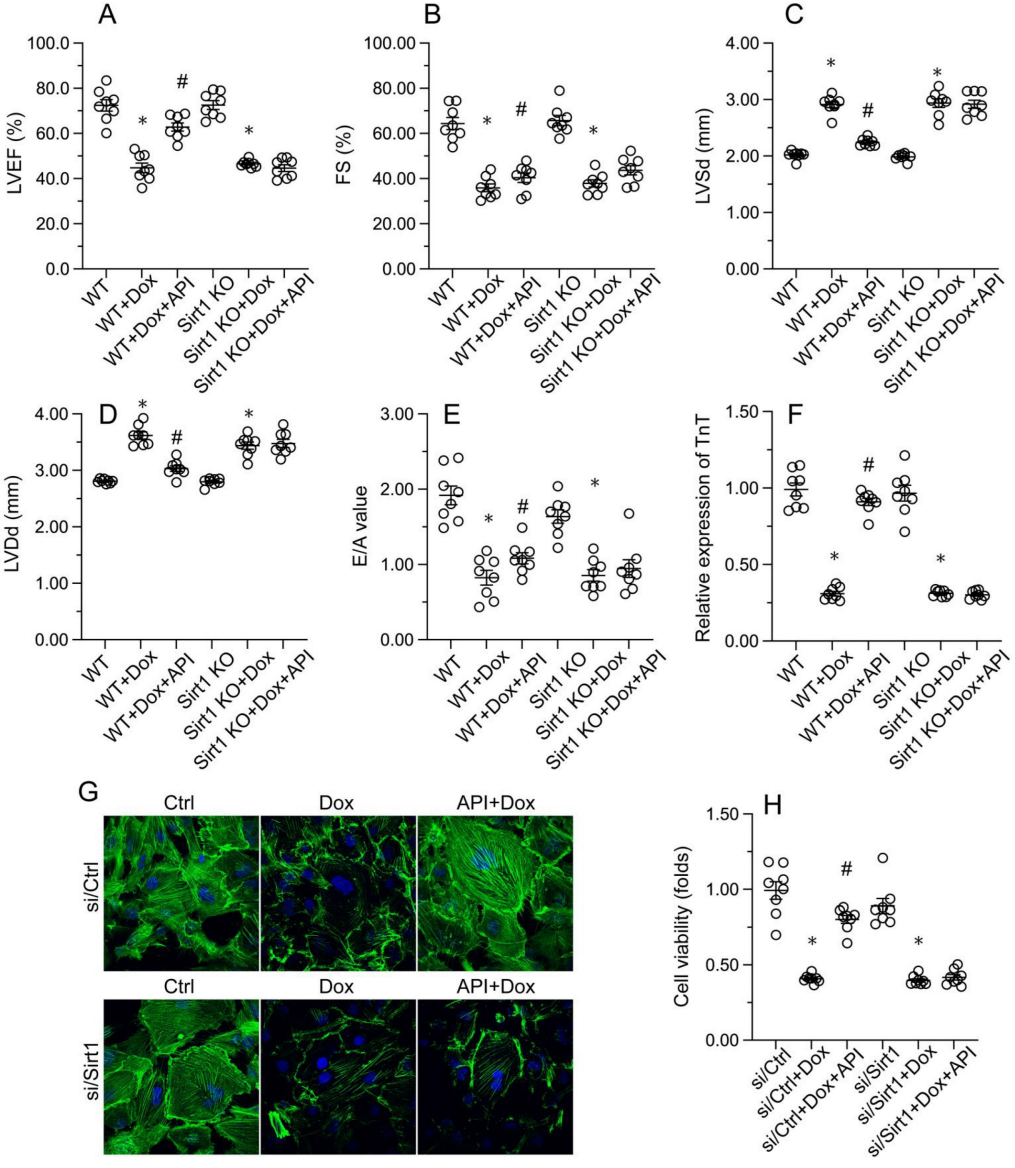
***Sirt1* knockdown abolishes the cardioprotective effects of apigenin in mice with Dox-induced cardiomyopathy.** Wild-type (WT) and *Sirt1* knockout (KO) mice were treated with Dox (15 mg/kg) with or without concomitant oral apigenin (25 mg/kg) administration for 4 weeks. **(A-C)** Serum levels of TnT, BNP, and CK-MB were measured by ELISA. **(D)** Sirius Red staining was used to observe histological alterations in heart tissue. **(E**,** F)** GR-1 immunofluorescence was used to assess neutrophil infiltration in the myocardium.** (G-H)** RT-PCR was used to analyze the transcription of IL-6, MCP1, and MMP9 in heart tissues. *p<0.05.

To verify whether Sirt1/Atf5 pathway activation underlies apigenin-induced cardioprotection against Dox-induced cardiomyopathy, apigenin was co-administered to Dox-treated *Sirt1* knockout (KO) mice. Interestingly, the reduction in TnT, BNP, and CK-MB expression observed in Dox-treated WT mice was not evident in *Sirt1* KO mice (Figure 6A-6C). Similarly, Sirius Red staining showed that apigenin's ability to sustain cardiomyocyte structure and prevent Dox-mediated myofibrillar fibrosis was significantly attenuated in *Sirt1* KO mice (Figure 6D). Moreover, GR-1 immunofluorescence further revealed that apigenin failed to prevent Dox-mediated myocardial neutrophil accumulation (Figure 6E and 6F), as well as transcriptional upregulation of inflammation-related cytokines (Figure 6G-6H), in *Sirt1* KO mice. On the basis of the above findings, we concluded that Sirt1 deficiency abrogated the cardioprotective actions of apigenin on Dox-induced cardiomyopathy.

### Sirt1 silencing abrogates apigenin's ability to prevent Dox-mediated cardiomyocyte dysfunction

**Figure 7:**
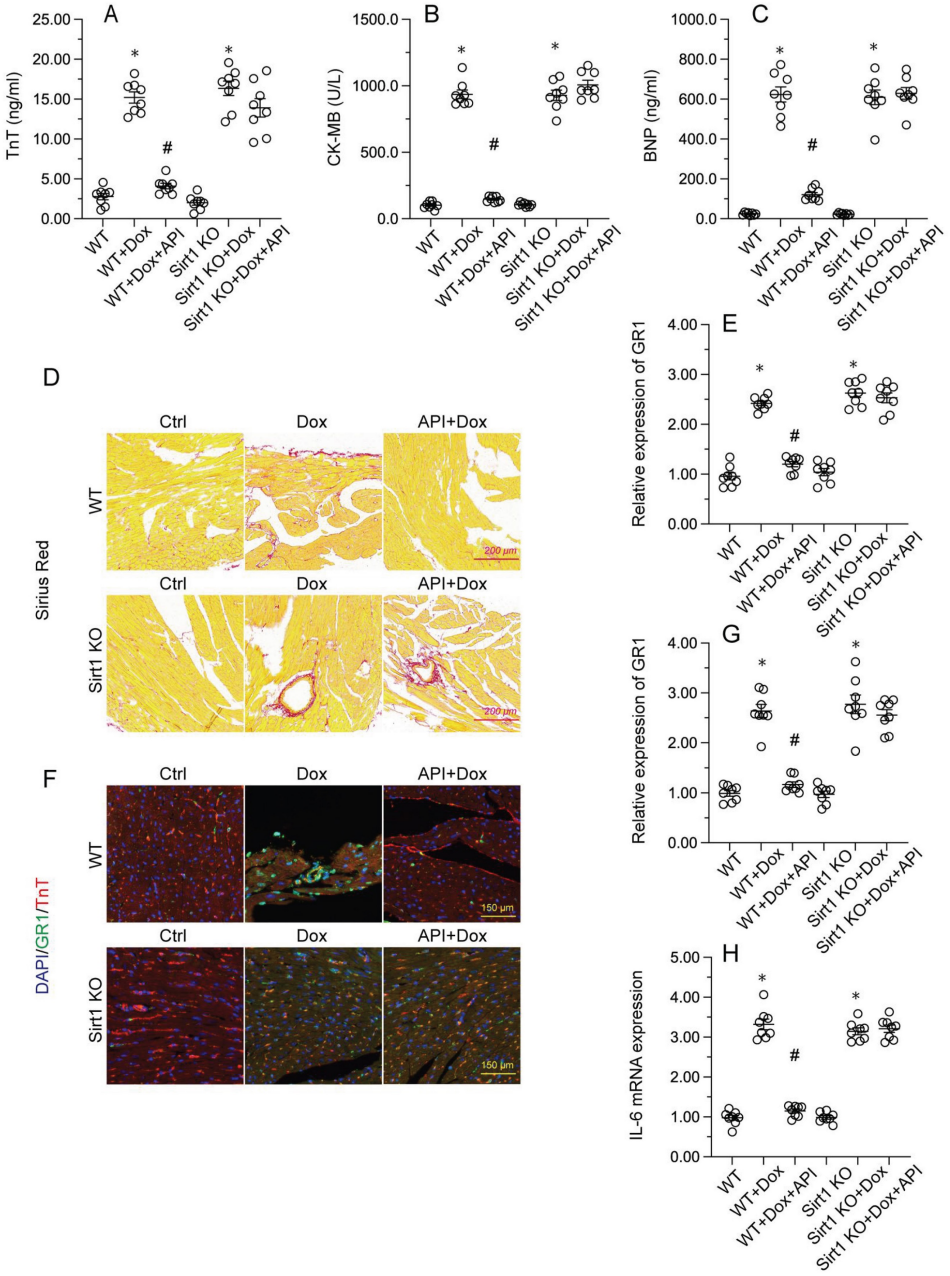
** Apigenin fails to preserve cardiomyocyte function after *Sirt1* deletion.** *In vivo,* **(A-E)** Echocardiographic analysis of heart function in WT and *Sirt1* KO mice. **(F**,** G)** Immunofluorescence detection of TnT in cultured HL-1 cells. **(H)** The MTT assay was used to analyze HL-1 cell viability. *p<0.05.

Consistent with the above findings, the beneficial effects of apigenin on heart function, observed in Dox-treated WT mice, were instead undetectable in *Sirt1* KO mice. This was evidenced by impaired LVEF, FS, LVSd, LVDd, and E/A in *Sirt1* KO relative to WT mice (Figure 7A-7E). Indicative of preserved contractile function, the expression of TnT was normalized by apigenin in Dox-challenged HL-1 cells (Figure 7F and 7G). However, apigenin failed to maintain TnT expression upon transfection of si/Sirt1 (Figure 7F and 7G). Further, MTT assays showed that the ability of apigenin to sustain cardiomyocyte viability in the presence of Dox was inhibited once infection with si/Sirt1 (Figure 7H). Our data confirmed that in the setting of on Dox-induced cardiomyopathy, Sirt1 deficiency weakens the cardioprotective actions of apigenin.

## Discussion

Our observation offers robust evidence for the cardioprotective actions of apigenin on Dox-induced cardiomyopathy. Importantly, our experiments demonstrated that activation of the UPR^mt^ is the primary molecular mechanism underlying apigenin-mediated protection against Dox-induced cardiotoxicity. On the one hand, pharmacological blockade of the UPR^mt^ abolished the protective effects of apigenin on cardiomyocyte viability and mitochondrial integrity. On the other hand, gene knockout assays conducted both *in vivo* and *in vitro* further revealed that apigenin activates the UPR^mt^ through the Sirt1/Atf5 pathway. Our findings hence support the therapeutic potential of apigenin in the treatment of Dox-induced cardiomyopathy, and highlight the Sirt1/Atf5/UPR^mt^ axis as a promising target for the design and development of drugs against Dox-related cardiotoxicity.

Apigenin is an effective active ingredient extracted from Ludangshen Oral Liquid. Recent animal studies have elucidated various cardioprotective actions of apigenin in cardiovascular disorders 31. In accordance with our data, recent experiments revealed the anti-apoptotic effects of apigenin-coated gold nanoparticles in a mouse model of Dox-induced cardiotoxicity 32. It was also reported that apigenin alleviated aluminum phosphide-induced cytotoxicity in rat cardiomyocytes 33. Administration of apigenin before myocardial infarction was found to reduce cardiomyocyte death by activating Parkin-dependent mitophagy 34. Similarly, in hypoxia-treated cardiomyocytes, apigenin was reported to activate the HIF-1a pathway and thus enhance glucolipid metabolism through restoring mitochondrial function 35. Nutritional supplementation of apigenin before induction of myocardial IRI protected mitochondrial integrity through a mechanism involving the Notch1/Hes1 pathway 36. Indeed, previous studies had uncovered important mitoprotective actions of apigenin in several disease models 37. For example, apigenin was reported to reduce palmitic acid-induced mitochondrial dysfunction through mitophagy activation, resulting in decreased hepatic pyroptosis 38. During LPS-caused neurotoxicity, apigenin was shown to activate mitochondrial fusion, an important mechanism safeguarding mitochondrial homeostasis, through a mechanism involving the Sirt3/PINK1/Parkin pathway 39. The potential anticancer activity of apigenin has also been proposed, based on reported induction of mitochondria-mediated caspase-9 activation and apoptosis in breast cancer cells 40. The above findings indicate that mitochondria are a key target of apigenin, and suggest that the cardioprotective actions of apigenin are closely associated with increased mitochondrial fitness.

The UPR^mt^ represents a mitochondria-controlled nuclear transcriptional system that upregulates the levels of mitoprotective genes to prevent oxidative injury, inflammation, and apoptosis 41-43. The cardioprotective effects of UPR^mt^ activation have been confirmed in rodent models of cardiac IRI 44. Specifically, evidence indicates that Atf5-dependent UPR^mt^ rescues mitochondrial metabolism and favors cardiomyocyte survival during hypoxic stress 44. An observation from Wang et al. highlighted a critical role of the UPR^mt^ in septic cardiomyopathy 8. They showed that pharmacological activation of the UPR^mt^ restored mitochondrial quality control, as evidenced by decreased mitochondrial fission, enhanced mitochondrial fusion, and normalized mitophagy, leading to attenuated inflammatory response and reduced cardiomyocyte death 8, 12, 45-47. Herein, we provide new data to support the regulatory action of apigenin on UPR^mt^, by identifying the Sirt1/Atf5 pathway as a central mechanism underlying apigenin's cardioprotective effects against Dox-induced cardiomyopathy. To data, this is the first study to elucidate the working mechanism through which apigenin activates the UPR^mt^ in cardiomyocytes 48. However, several questions remain to be answered regarding the regulatory actions of apigenin on UPR^mt^. First, although Atf5 is regarded as an UPR^mt^ inducer, the mechanism by which Atf5 triggers this homeostatic response remains unclear. Second, although pharmacological inhibition of the UPR^mt^ with ABESF allowed us to confirm that apigenin-mediated UPR^mt^ activation is linked to improved mitochondrial performance, the molecular interactions underlying this outcome were not specifically investigated.

Accumulating evidence has identified Sirt1 as an important intracellular sensor of cardiomyocyte damage and mitochondrial dysfunction in Dox-induced cardiotoxicity. During Dox-induced cardiomyopathy, pharmacological activation of Sirt1 was found to upregulate Nrf2 expression and thus inhibit oxidative stress and ferroptosis in cardiomyocytes 49, 50. Notably, in the setting of Dox-related cardiomyopathy, increased Sirt1 expression is closely associated with enhanced PGC1α activity, which promotes mitochondrial biogenesis and fatty acid oxidation, resulting in improved cardiomyocyte metabolism, decreased myocardial fibrosis, and restored heart function 51. NF-κB has been identified as a downstream target of Sirt1. Interestingly, Sirt1 upregulation prevented NF-κB activation and thus suppressed inflammation in a rat model of Dox-induced cardiotoxicity 52. In our study, we found that Sirt1 activation promotes the expression of Atf5, an inducer of the UPR^mt^. This evidence enriches our understanding of the molecular network controlled by Sirt1 in the setting of Dox-induced cardiomyopathy 53, 54. Importantly, our results, in combination with previous findings, further confirmed the central role of Sirt1 in protecting the heart against Dox-mediated injury 11. Nevertheless, several limitations need to be addressed in future studies. First, the mechanism by which apigenin influences Sirt1 activity has not been clarified, and further research is needed to assess whether transcriptional or post-transcriptional mechanisms are involved. Second, from a clinical standpoint, it would be interesting to evaluate whether apigenin effectively attenuates myocardial injury once early symptoms of Dox-induced myocardial cardiotoxicity have manifested.

In sum, our work revealed that apigenin exerts therapeutic effects in mice with Dox-mediated cardiomyopathy through activation of the Sirt1/Atf5/UPR^mt^ axis in cardiomyocytes. Our data suggest that apigenin upregulates the expression of Sirt1 and therefore enhances Atf5 levels, resulting in increased UPR^mt^ activity. During Dox exposure, either knockdown of Sirt1 or UPR^mt^ blockade neutralized the beneficial effects of apigenin, as evidenced by defective mitochondrial function and increased cardiomyocyte death. Our findings thus suggest that apigenin constitutes a promising therapeutic alternative to protect heart function in patients treated with Dox.

## Materials and Methods

### Animal model and cell treatment

Dox-induced cardiomyopathy was modeled in wild type (WT) and *Sirt1* knockout (*Sirt1*-KO) mice (strain #026009, The Jackson Laboratory) by administration of Dox (15 mg/kg/d) for 4 weeks. Independent groups of WT and *Sirt1*-KO mice received, in addition to Dox, apigenin (API; 25 mg/kg/d) for 4 weeks via gavage. Control mice received only PBS. Thus, 5 mouse groups were established: 1) PBS control (n = 6); 2) WT DOX (n = 6); 3) WT DOX + API (n = 6); 4) *Sirt1*-KO DOX (n = 6); and 5) *Sirt1*-KO DOX + API (n = 6).

To mimic Dox-induced cardiomyopathy *in vitro*, mouse HL-1 cardiomyocytes maintained in DMEM and were treated with 1 μM Dox for 24 h. Separate cell cultures were co-incubated, along with 1 μM Dox, with 50 μM apigenin. To inhibit the mitochondrial unfolded protein response, ABESF (10 μM, #78431, Thermo Fisher) was added to cultured HL-1 cells 4 h before Dox/apigenin addition.

### Histology and immunofluorescence

Samples were dissected and fixed with 4% paraformaldehyde. Five-µm cross-sections of paraffin-embedded heart samples were stained with Sirius Red. For GR-1 (a neutrophil marker) and cytochrome c immunostaining, frozen heart sections were fixed in 10% formalin. Sections were treated overnight at 4°C with antibodies against GR-1 (Abcam, ab25377) or cytochrome c (Abcam, ab76107) diluted in 1% BSA in PBS-Tween 20 (0.1%) 55. The sections were subsequently counterstained with DAPI, mounted, and examined using the Nikon Ni-U microscope (Nikon Eclipse Ni-U) 56. Images were captured using an RT3 Slider digital camera system (WS-RT2540-0480, Diagnostic Instruments, Inc.) 57.

### Transthoracic echocardiography

Myocardial performance was evaluated using a Vevo2100 Ultrasound system (VisualSonics, Toronto, Canada) at various post-surgery time points 58, 59. Two-dimensional B-Mode imaging was used to observe the left ventricle and the aortic outflow tract. M-Mode echocardiographic images were recorded by placing the sample line at the maximum cross-section of the left ventricle 60.

### MTT-based cell viability assay

HL-1 cells were seeded in 6-well plates and transfected with test siRNAs (see below) for 48 h. The cells were then transferred to 96-well flat bottom plates (50,000 cells per well) before further incubation overnight. Ten μL of MTT (3-(4,5-dimethylthiazol-2-yl)-2,5-diphenyltetrazolium bromide) solution, provided in the MTT Cell Growth Assay Kit (#CT01, Millipore), was then added to the culture medium of each well for 4-h incubation at 37°C. The formazan formation was detected colorimetrically by using 100 μL acidic isopropanol 61.

### Cellular transfections

Cells were transfected with control siRNA (sc-37007), Sirt1 siRNA (sc-40987), or Atf5 siRNA (sc-60222) purchased from Santa Cruz Biotechnology, in combination with Lipofectamine RNAiMAX Reagent (13778150, Invitrogen) following the manufacturer's protocol 50.

### Western blots and RNA extraction and real-time polymerase chain reaction (RT-PCR)

Western blots were performed as our previously described. Sirt1 (Cat. No. ab110304, Abcam) or Atf5 (Cat. No. ab184923, Abcam), and GAPDH (Cat. No. ab8245, Abcam) were used in our study. RNA isolation was performed using TRIzol reagent (Invitrogen). Subsequently, a Reverse Transcription Reagent kit was used to reverse-transcribe RNA into cDNA which cDNA was then amplified by semi-quantitative RT-PCR using SYBR Green Mix. The primers used for RT-PCR are listed in Supplementary Table 1.

### Enzyme-linked immunosorbent assay (ELISA)

ELISAs were performed as recommended by the manufacturer using mouse serum or cell culture supernatants. Signals were measured using a Tecan Infinite 200 Pro (Tecan, Zürich, Switzerland) instrument. The following ELISA kits were used: ATP concentration (LS-F24998, LifeSpan BioSciences), serum Troponin T (TNT) concentration (abx496644, Abbexa), serum BNP concentration (NBP2-70011, Novus), serum CK-MB concentration (NBP2-75312, Novus), Gpx activity (ab102530, Abcam), Prx3 activity (ab277720, Abcam), and Txnrd2 activity (MBS9328396, MybioSource) 10.

### Statistical Analysis

The data are presented as mean ± SE. Statistical analyses were conducted using GraphPad Prism. ANOVA followed by Tukey's post hoc test was used for multiple comparisons, unless otherwise specified. In cases where only two groups were compared, a two-tailed Student's t-test with unequal variance was performed. Statistical significance was defined as p < 0.05.

## Supplementary Material

Supplementary table.
