# Supplementary material for: Screening of an FDA-approved compound library identifies apigenin for the treatment of myocardial injury
Source: Int J Biol Sci. 2023 Oct 16;19(16):5233–44. doi: 10.7150/ijbs.85204 (PMC10620826; doi:10.7150/ijbs.85204)
Supplement: Supplementary file 1 — Supplementary table. [file ijbsv19p5233s1.pdf]

Supplemental Table 1: Primer sequences for RT-PCR

Gene	Forward (5'-3')	Reverse (5'-3')
**CHOP (mouse)**	GCGACAGAGCCAGAATAACA	GCGACAGAGCCAGAATAACA
**mtDNAj (mouse)**	AGTCACCCACACAAGCACTG	CCAGCCTCTCGCCTATCC
**ClpP (mouse)**	CACAGACATCGCCATCCA	TCCCTCTCCATTGCTGACTC
**LonP1 (mouse)**	GGTTGAGAATGTAGCCCATGA	CGATGATATCCCGAATGGTC
**Atf5 (mouse)**	TCCGCTCACACCGTCTCT	AAGGCGAAGGTGGAGGAC
**Hsp10 (mouse)**	GGCCCGAGTTCAGAGTCC	TGTCAAAGAGCGGAAGAAACTT
**MMP9 (mouse)**	CTTCTGGCGTGTGAGTTTCCA	ACTGCACGGTTGAAGCAAAGA
**IL-6 (mouse)**	CAGACTCGCGCCTCTAAGGAGT	GATAGCCGATCCGTCGAA
